# Immunotherapy After Chemotherapy and Radiation for Clinical Stage III Lung Cancer

**DOI:** 10.1001/jamanetworkopen.2022.24478

**Published:** 2022-08-04

**Authors:** Matthew D. Pichert, Maureen E. Canavan, Richard C. Maduka, Andrew X. Li, Theresa Ermer, Peter L. Zhan, Michael Kaminski, Brooks V. Udelsman, Justin D. Blasberg, Henry S. Park, Sarah B. Goldberg, Daniel J. Boffa

**Affiliations:** 1 Division of Thoracic Surgery, Department of Surgery, Yale University School of Medicine, New Haven, Connecticut; 2 Cancer Outcomes Public Policy and Effectiveness Research Center, Department of Internal Medicine, Yale University School of Medicine, New Haven, Connecticut; 3 London School of Hygiene and Tropical Medicine, University of London, London, United Kingdom; 4 Department of Therapeutic Radiology, Yale School of Medicine, Smilow Cancer Hospital at Yale, New Haven, Connecticut; 5 Section of Medical Oncology, Department of Internal Medicine, Yale School of Medicine, New Haven, Connecticut

## Abstract

**Importance:**

The 2017 international PACIFIC trial established a role for immunotherapy after chemoradiation for unresectable stage III non–small cell lung cancer (NSCLC). However, in the US, patients with NSCLC commonly differ from clinical trial populations in terms of age, health, access to care, and treatment course, which may all factor into the efficacy of immunotherapy.

**Objective:**

To determine the outcomes of immunotherapy use in unresectable stage III NSCLC in the general US population.

**Design, Setting, and Participants:**

This cohort study analyzed the National Cancer Database for patients diagnosed with clinical stage III NSCLC between 2015 and 2017 with follow-up through the end of 2018 who were treated with chemotherapy and radiation. Data were analyzed January 2022.

**Main Outcomes and Measures:**

Mortality hazard in a multivariable Cox proportional hazards model and survival among a propensity-matched sample treated with chemotherapy and radiation, with and without immunotherapy.

**Results:**

A total of 23 811 patients with clinical stage III NSCLC with median (IQR) age 66 (59-72) years met inclusion criteria (10 454 [43.9%] women; 564 [2.4%] Asian, 2930 [12.3%] Black, 20 077 [84.3%] White patients), including 209 (16.1%) patients with multiple comorbidities and 1297 (5.4%) immunotherapy recipients. Immunotherapy after chemotherapy and radiation was associated with reduced mortality (hazard ratio [HR], 0.74; 95% CI, 0.67-0.82; *P* < .001). Among a propensity-matched sample, immunotherapy was associated with superior 3-year survival (52% [1297 patients at 0 months, 56 patients at 36 months] vs 44% [2594 patients at 0 months, 173 patients at 36 months]; *P* < .001). The treatment of 833 patients who received immunotherapy (64.2%) differed from the PACIFIC trial protocol, including 221 patients (17.0%) who received radiation doses outside of the protocol range and 731 patients (56.4%) who started immunotherapy more than 6 weeks after radiation was completed. The survival advantage of immunotherapy persisted when initiated up to 12 weeks after radiation was completed (HR, 0.75; 95% CI, 0.61-0.92). Among patients who received radiation outside the PACIFIC protocol range, the survival advantage of immunotherapy was not significant (HR, 0.87; 95% CI, 0.69-1.01).

**Conclusions and Relevance:**

In this cohort study, immunotherapy after chemotherapy and radiation for stage III NSCLC was associated with a survival advantage in the general US population despite two-thirds of patients treated differently than the PACIFIC protocol. The findings suggest there may be flexibility in the timing of immunotherapy initiation after radiation; further study is warranted to clarify the clinical benefits of immunotherapy.

## Key Points

**Question:**

Is immunotherapy after chemotherapy and radiation for stage III unresectable non–small cell lung cancer (NSCLC) associated with a survival advantage in the US, particularly when care differs from trial protocols?

**Findings:**

In this cohort study, immunotherapy was associated with a survival advantage among clinical stage III NSCLC patients, including patients treated outside the landmark PACIFIC trial protocol.

**Meaning:**

These results suggest that immunotherapy benefits for stage III NSCLC may extend to the general population in the US and there may be flexibility in timing of immunotherapy initiation.

## Teaser

This cohort study of US adults in the National Cancer Database compares outcomes for patients with unresectable stage III non–small cell lung cancer treated with immunotherapy after chemotherapy and radiation with those treated only with chemotherapy and radiation.

## Introduction

Approximately 35 500 patients are diagnosed with stage III non–small cell lung cancer (NSCLC) in the US each year. Stage III NSCLC can be particularly difficult to manage, as evidenced by the 5-year survival rates hovering around 20%.^1,2^ In 2017, an international clinical trial (PACIFIC trial) demonstrated a 17.2% improvement in 18-month progression-free survival when immunotherapy, specifically the anti–programmed death ligand 1 (anti–PD-L1) antibody durvalumab, was administered after definitive chemoradiation to patients with unresectable stage III NSCLC.^3,4,5,6^ In February 2018, the US Food and Drug Administration approved the use of durvalumab after chemoradiation for unresectable stage III NSCLC, thereby establishing the role of immunotherapy as the standard of care for this patient population.^7,8^

The benefits of innovative cancer treatments in the general population may differ from those achieved during clinical trials. Classically, cancer trials have focused on healthier patients in an attempt to minimize potential confounding effects that medical comorbidities could have on the observed results.^9^ In fact, only 20% to 35% of patients with NSCLC meet eligibility criteria for the clinical trials designed for their disease status.^10^ Furthermore, patients in the general population may face logistical challenges or socioeconomic disadvantages that impair their ability to comply fully with intended treatment or follow-up appointments.^11^ Considering that compliance with treatment and cancer-related outcomes have been associated with payer status, this differential may be particularly important in the US, where there is a lack of universal health coverage.^12,13^ Finally, care plans tend to deviate more frequently in the general population compared with the clinical trial setting.^14,15^ As a result, there is more variability in the treatment received, which could affect treatment efficacy. A better understanding of outcomes associated with immunotherapy outside of the clinical trial setting could not only give a more accurate clinical perspective but potentially reveal opportunities to be more flexible with treatment regimens, improving patient compliance.

The National Cancer Database (NCDB) captures the care of approximately 70% of newly diagnosed lung cancers in the US.^16,17^ Given the NCDB’s large catchment with prospectively collected outcomes over multiple decades, the NCDB is uniquely equipped to evaluate care outside of the clinical trial setting in the US.

In this retrospective cohort study, we evaluated patients with clinical stage III NSCLC treated with chemotherapy and radiation in the NDCB to assess the survival associated with the addition of immunotherapy. Our objective was to understand the outcomes associated with this evolving standard of care within the general population of the US, and to examine differences in outcomes associated with variations in treatment regimen and timing.

## Methods

### Database

The NCDB is a joint venture of the Commission on Cancer and the American Cancer Society, which prospectively captures tumor, demographic, treatment, and outcome data on nearly 70% of newly diagnosed cases of cancer within the US.^16,17^ This study was conducted in compliance with our protocol approved by the Yale School of Medicine institutional review board, with informed consent requirements waived because data were deidentified. The Strengthening the Reporting of Observational Studies in Epidemiology (STROBE) reporting guideline for cohort studies was followed in the creation of this manuscript.

### Participant Selection

The 2018 NCDB participant user file was queried for patients aged 18 years or older who were diagnosed with clinical stage III NSCLC from 2015 to 2017 and had received multiagent chemotherapy and at least 45 Gy of radiation. Patients were excluded if follow-up data, immunotherapy treatment status, or vital status were missing (3622 patients excluded) (eTable 2 in the Supplement). A sensitivity analysis was performed between included and excluded patients; no obvious clinically important differences were noted (data available upon request).

### Covariates

The following independent variables were studied: age (stratified as 20 to 49 years, 50 to 64 years, 65 to 75 years, and 75 years and older in Cox models), sex, race (Asian, Black, White, other), ethnicity (Hispanic, non-Hispanic), comorbidities represented as modified Charlson-Deyo score, histology, clinical T category, clinical N category, payer status (uninsured, private, Medicare, Medicaid), facility type (academic, nonacademic), facility location (Northeast, Midwest, South, West), and median household income as determined by the zip code in which the patient lived during the year of diagnosis. Race and ethnicity data from patient medical records were included as covariates as these variables can be surrogates for social determinants of health. The full NCDB data dictionary is available online.^18^

### Statistical Analysis

#### Missing Data Strategy

Overall, the rates of missing data were low: less than 3% for all variables except zip code–level median income, which was missing for 13% of patients (Table). Reviewing the patterns of missing data did not identify any indication that data was not missing at random; however, we attempted to account for all variables that might be correlated with missing data for covariates of interest in our multiple imputation model and achieved similar results for our Cox models using complete cases and multiple imputation analyses via chained equations.^19,20^ Rubin rules were used to generate pooled effect estimates and variance across imputed data sets.^20^ A complete case approach was performed as a sensitivity analysis, which achieved similar results (data available on request).

**Table.** Bivariate Analysis of Patients With Stage III NSCLC Treated Definitively With Chemotherapy and Radiation With or Without Immunotherapy

Characteristics	Patients, No. (%)^a^		*P* value
	Chemoradiation only	Chemoradiation followed by immunotherapy	
Total patients	22 514 (94.6)	1297 (5.4)	
Age, y			
20-49	827 (3.7)	55 (4.2)	.01
50-64	8455 (37.6)	519 (40.0)	
65-74	8645 (38.4)	509 (39.2)	
≥75	4587 (20.4)	214 (16.5)	
Sex			
Men	12 659 (56.2)	698 (53.8)	.09
Women	9855 (43.8)	599 (46.2)	
Race			
Asian	528 (2.4)	36 (2.8)	.65
Black	2778 (12.3)	152 (11.7)	
White	18 983 (84.3)	1094 (84.4)	
Other/unknown^b^	225 (1.0)	15 (1.2)	
Ethnicity			
Hispanic	506 (2.3)	29 (2.2)	.96
Non-Hispanic	21 583 (95.9)	1245 (96.0)	
Unknown	425 (1.9)	23 (1.8)	
Insurance			
Private	6403 (28.4)	413 (31.8)	.07
Medicare	12 831 (57)	689 (53.1)	
Medicaid	1951 (8.7)	124 (9.6)	
Uninsured	487 (2.2)	29 (2.2)	
Other government	556 (2.5)	27 (2.1)	
Unknown	286 (1.3)	15 (1.2)	
Median income, $			
<38 000	3929 (17.5)	206 (15.9)	.06
38 000-47 999	5042 (22.4)	285 (22.0)	
≥48 000	10 516 (46.7)	600 (46.3)	
Unknown	3027 (13.4)	206 (15.9)	
Year of diagnosis			
2015	7905 (35.1)	66 (5.1)	<.001
2016	7926 (35.2)	78 (6.0)	
2017	6683 (29.7)	1153 (88.9)	
Charlson-Deyo Comorbidity score			
0	13 476 (59.9)	748 (57.7)	.40
1	5709 (25.4)	340 (26.2)	
2	2131 (9.5)	131 (10.1)	
3	1198 (5.3)	78 (6.0)	
Histology			
Adenocarcinoma	9922 (44.1)	591 (45.6)	.16
Squamous cell	10 293 (45.7)	581 (44.8)	
Large cell	332 (1.5)	10 (0.8)	
Other	1967 (8.7)	115 (8.9)	
T category			
0	100 (0.4)	0	.01
1	4428 (19.7)	223 (17.2)	
2	6631 (29.5)	374 (28.8)	
3	5373 (23.9)	327 (25.2)	
4	5640 (25.1)	359 (27.7)	
Unknown	342 (1.5)	14 (1.1)	
N category			
0	1771 (7.9)	86 (6.6)	<.001
1	1612 (7.2)	95 (7.3)	
2	14 438 (64.1)	780 (60.1)	
3	4614 (20.5)	334 (25.8)	
Unknown	79 (0.4)	2 (0.2)	
Stage			
IIIA	15 225 (67.6)	780 (60.1)	<.001
IIIB	7221 (32.1)	517 (39.9)	
Other (III, IIIC)	68 (0.3)	0	
Facility type			
Nonacademic	15 375 (68.3)	834 (64.3)	.01
Academic	7046 (31.3)	458 (35.3)	
Unknown	93 (0.4)	5 (0.4)	
Region			
Northeast	4576 (20.3)	254 (19.6)	.69
Midwest	6921 (30.7)	417 (32.2)	
South	8423 (37.4)	468 (36.1)	
West	2501 (11.1)	153 (11.8)	
Unknown	93 (0.4)	5 (0.4)	
90-d mortality			
Yes	5 (0.1)	0	.67
No	10 472 (100)	858 (100)	
Clinical trial participation			
No	22 456 (99.7)	1284 (99.0)	<.001
Yes	58 (0.3)	13 (1.0)	
Radiation dosing, Gy			
45-53 (<PACIFIC dose)	4163 (18.5)	158 (12.3)	<.001
54-59	1611 (7.2)	94 (7.3)	
60 (middle PACIFIC dose)	9605 (42.7)	653 (50.9)	
61-66	6014 (26.7)	329 (25.7)	
≥67 (>PACIFIC dose)	1121 (5.0)	63 (4.9)	
Time to immunotherapy, wk^c^^,^^d^			
≤6	NA	551 (43.0)	NA
7-9	NA	328 (25.6)	
10-12	NA	178 (13.9)	
≥13	NA	225 (17.6)	

^a^ Percentages may not add to 100 due to rounding.

^b^ Other includes patients identified as other or unknown race in the National Cancer Database.

^c^ *P* values were generated comparing immunotherapy to chemotherapy and radiation only.

^d^ Time from end of radiation treatment to starting immunotherapy.

#### Landmarking for Immortal Time Bias

Survival was determined in days from diagnosis. Given that immunotherapy is administered after completion of radiation, there is a potential for immortal time bias.^21^ For the primary adjusted models, landmarking was performed at the maximal time noted by the PACIFIC trial (6 weeks). For the time-to-initiation analyses, patients were stratified into subgroups based on 3-week intervals of immunotherapy initiation and landmarked at the middle week (eg, the analysis of the cohort beginning immunotherapy 7 to 9 weeks after start of radiation was landmarked at 8 weeks). Total radiation was calculated as the sum of the initial therapy and all subsequent boost doses of radiation listed in the NCDB.

Univariable analyses were performed between the patients who received chemotherapy and radiation vs those who received immunotherapy in addition to chemotherapy and radiation. A Kaplan-Meier analysis was performed for the above defined groups, and a Cox proportional hazards model was created with the above defined groups that included covariates such as age, race, sex, ethnicity, zip code level median income, insurance status, Charlson-Deyo comorbidity score, histology, clinical N category, clinical T category, facility type, region, and year of diagnosis. Violations of the proportional hazards assumption were assessed using Martingale residuals. No violations were detected. A propensity match was performed between the above defined groups in a 1 to 2 fashion using the following covariates: age group, sex, race, ethnicity, insurance status, zip code–level median income, sex, Charlson-Deyo comorbidity score, histology, clinical N stage, clinical T stage, facility type, region, and year of diagnosis with standardized differences less than 0.1 for all pairs (eTable 1 in the Supplement). Clustering was accounted for including a clustering term for facility identification. To account for potential false positive associations within our stratified models we applied the Benjamini-Hochberg procedure to significant associations using a false discovery rate of 0.05.^22^

A Kaplan-Meier analysis was performed on the propensity matched groups. All statistical analyses were conducted using SAS version 9.4 (SAS Institute) and 2-sided *P* values < .05 were considered significant.

## Results

### Patients

Overall, 23 811 patients with clinical stage III NSCLC who received chemotherapy and radiation were identified (median [IQR] age, 66 [59-72] years; 10 454 [43.9%] women; 564 [2.4%] Asian, 2930 [12.3%] Black, 20 077 [84.3%] White patients), including 1297 (5.4%) who received immunotherapy. A total of 214 (16.5%) of patients receiving immunotherapy were older than 75 years of age and 153 (11.8%) were either uninsured or on Medicaid. Of the 1297 patients who received immunotherapy, 209 (16.1%) had a Charlson-Deyo Comorbidity Index score of 2 or 3. The use of immunotherapy increased over time (from less than 1% of patients with stage III NSCLC in 2015 to 15% patients in 2017) (Table). A total of 833 (64.2%) patients receiving immunotherapy were treated in a manner that differed from the PACIFIC protocol. More specifically, the PACIFIC trial dictated that immunotherapy started within 42 days of the radiation end date.^5^ In this study, 731 patients (56.4%) started immunotherapy more than 42 days after radiation had ended (Table). In addition, the PACIFIC protocol dictated that patients receive between 54 and 66 Gy of radiation.^5^ Overall, 158 (12.2%) of patients receiving immunotherapy received a lower dose than the PACIFIC range and 63 (4.9%) received a higher dose of radiation.

### Unadjusted Survival

A Kaplan-Meier survival analysis with 22 514 patients receiving chemotherapy and radiation only and 1297 patients receiving immunotherapy was performed with a median (IQR) follow-up of 32.4 (23.9-42.3) months among surviving patients (eFigure 1 in the Supplement). The addition of immunotherapy was associated with improved 3-year survival (52% [1297 patients at 0 months, 56 patients at 36 months] vs 44% [22 514 patients at 0 months, 5414 patients at 36 months]; P < .001).

### Adjusted Survival

**Figure 1.**
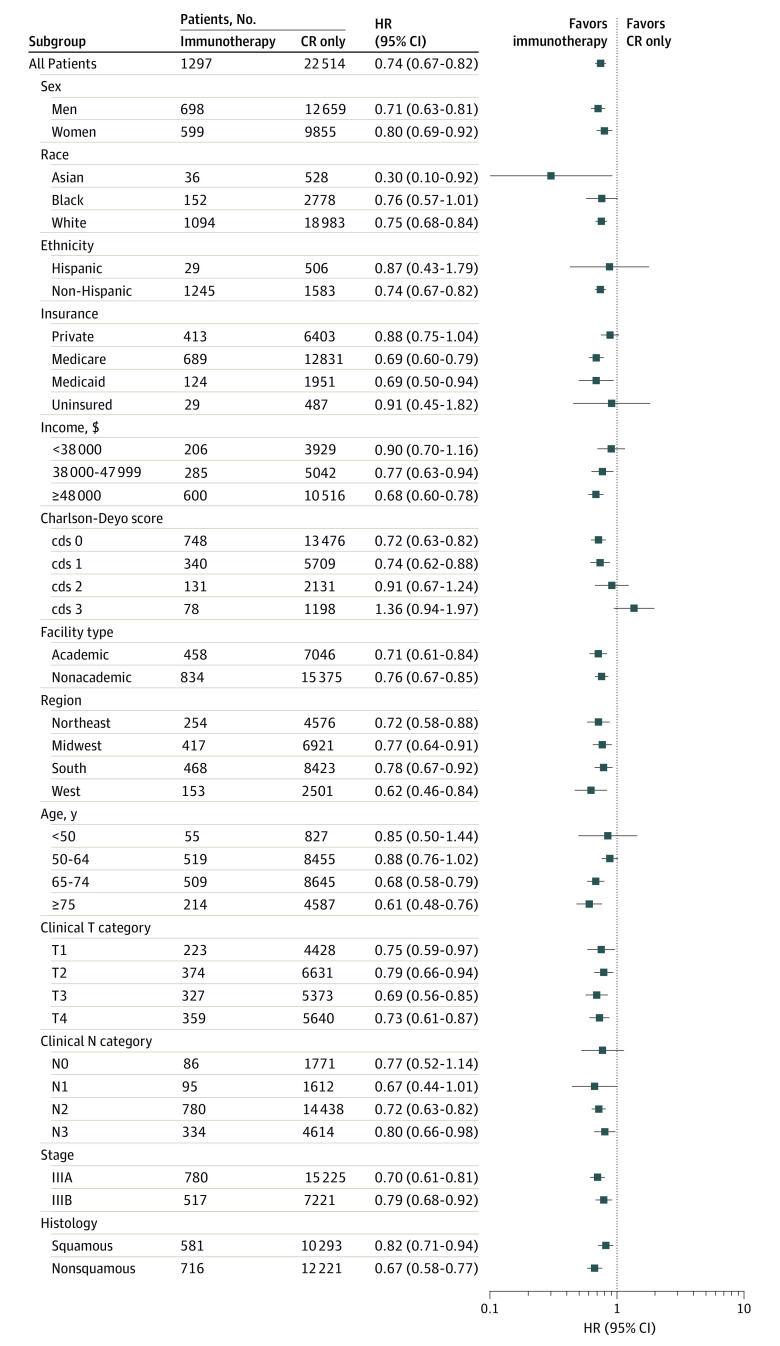
Forest Plot of Cox Proportional Hazard Models Stratified by Patient Attributes CR indicates chemotherapy and radiation; HR, hazard ratio. Significant associations were tested using the Benjamini-Hochberg procedure.

A multivariable Cox proportional hazards model was created and identified a number of factors associated with survival from a total of 23 811 patients (eTable 3 in the Supplement). The administration of immunotherapy was associated with a reduction in mortality (HR, 0.74; 95% CI, 0.67-0.82; *P* < .001). To examine the comparative effectiveness of immunotherapy across different patient, tumor, and treatment attributes, separate Cox models were created and stratified by each characteristic from a total of 1297 patients receiving immunotherapy and 22 514 patients receiving chemotherapy and radiation only (Figure 1). Immunotherapy was associated with a survival advantage across most of the stratified cohorts, including patients aged 75 years and older and those on Medicare or Medicaid.

**Figure 2.**
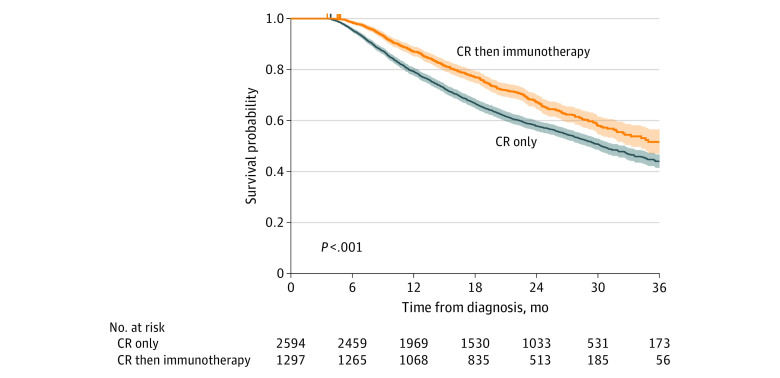
Kaplan-Meier Curve of Propensity Matched Patients With Stage III Non–Small Cell Lung Cancer Blue line indicates patients who received chemotherapy and radiation only; orange line, patients who received chemotherapy and radiation followed by immunotherapy; CR, chemotherapy and radiation.

In order to evaluate adjusted survival in a different way, propensity score matching was performed in a 2:1 fashion between the patients who did and those who did not receive immunotherapy after chemotherapy and radiation that included a total of 1297 patients receiving immunotherapy and 22 514 receiving chemotherapy and radiation (eTable 1 in the Supplement). A Kaplan-Meier analysis of propensity matched patients was performed with a median (IQR) follow-up of 25.5 (19.8-30.5) months among surviving patients (Figure 2). The 3-year survival was higher in the immunotherapy group compared to patients treated with chemotherapy and radiation alone, 52% (1297 patients at 0 months, 56 patients at 36 months) vs 44% (2594 patients at 0 months, 173 patients at 36 months) (*P* < .001).

### Outcomes Among Patients Whose Treatment Differed From PACIFIC Protocol

**Figure 3.**
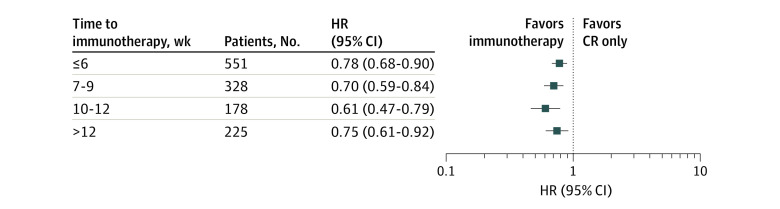
Forest Plot of Cox Proportional Hazard Models for Differing Amounts of Time From the End Date of Radiation to the Beginning of Immunotherapy CR indicates chemotherapy and radiation; HR, hazard ratio. Significant associations were tested using the Benjamini-Hochberg procedure. The time to immunotherapy window used in the PACIFIC trial was within 6 weeks.

The PACIFIC trial dictated that immunotherapy be initiated within 6 weeks of completing radiation. Overall, 1282 patients (56.3%) in the unmatched population study started immunotherapy beyond this time frame. Cox models were performed across a range of immunotherapy starting points for a total of 1297 patients receiving immunotherapy (Figure 3). Immunotherapy was associated with a survival advantage when given up to 12 weeks after the completion of radiation (HR, 0.75; 95% CI, 0.61-0.92).

Having explored whether or not immunotherapy offered an advantage across different time points for immunotherapy initiation, a separate analysis was performed to examine whether a superior time point for initiation could be identified out of the 1297 patients (eTable 7 in the Supplement). A Cox model of patients only receiving immunotherapy was created to compare different immunotherapy initiation time points against each other, including times within and outside of the PACIFIC trial protocol. No initiation time point appeared to be superior.

**Figure 4.**
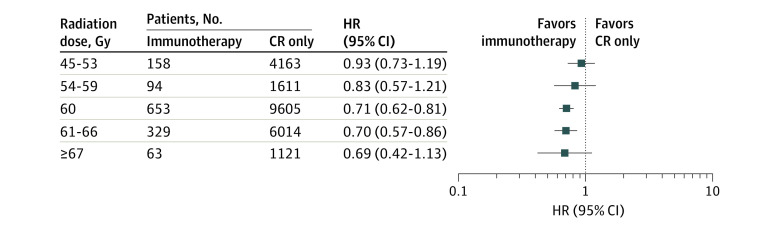
Forest Plot of Cox Proportional Hazard Models at Different Radiation Doses CR indicates chemotherapy and radiation; HR, hazard ratio. Significant associations were tested using the Benjamini-Hochberg procedure. The window for radiation doses used in the PACIFIC trial was between 54 and 66 Gy.

Radiation dosing was variable across the stage III NSCLC population, including 221 of 1297 patients receiving immunotherapy (17.0%) who were treated outside of the PACIFIC trial protocol. Separate Cox models were performed across radiation doses over a total of 1297 patients receiving immunotherapy and 22 514 receiving chemotherapy and radiation only (Figure 4). The benefit of immunotherapy (compared with chemotherapy and radiation alone) was only significant for patients treated with 60 Gy (HR, 0.71; 95% CI, 0.62-0.81) and 61 to 66 Gy (HR, 0.70; 95% CI, 0.57-0.86). It is important to note that 60 Gy was by far the largest subset (653 [50.9%] of patients received 60 Gy).

A separate Cox model of 1297 patients receiving immunotherapy compared different radiation doses with each other, both within and outside of the PACIFIC trial protocol range, to determine if a particular dose offered an advantage. No radiation dose was identified as being superior to the others (eTable 5 in the Supplement).

## Discussion

The current findings suggest that in the US, the addition of immunotherapy after chemotherapy and radiation for clinical stage III NSCLC resulted in improved overall survival compared with chemotherapy and radiation alone (HR, 0.74; 95% CI, 0.67-0.82). These findings support the PACIFIC trial findings. The magnitude of the overall survival benefit of immunotherapy is similar to the mortality reduction identified in the PACIFIC trial (HR, 0.68; 95% CI, 0.47-0.997).^4^ Several well-executed population studies have successfully replicated the PACIFIC trial findings in various parts of the world, including a study from the US Department of Veterans Affairs, with overall survival HRs ranging from 0.56 to 0.68 (eTable 8 in the Supplement), which demonstrates a degree of variability in possible outcomes.^23,24,25^

This population study extends the PACIFIC clinical trial findings by associating immunotherapy with a survival benefit in a cohort that included a wide range of patient age (16% were older than 75 years), health (16% of patients had multiple medical comorbidities), and socioeconomic status (24.5% had indicators of socioeconomic disadvantage). The initial PACIFIC report raised the question of diminished benefit of immunotherapy among older patients (ie, those aged 65 years and older).^5^ The current study found the survival advantage of immunotherapy persists in patients 75 years or older, consistent with a subsequent post hoc analysis in the PACIFIC cohort.^26^ Our results also confirmed an advantage of immunotherapy among a cohort that included a range of payers (12% had Medicaid or were uninsured), which is important as payer status has been associated with both the delivery of care and potentially the outcomes of cancer treatment.^27,28^

The majority of patients in this study (65%) were treated differently than in the PACIFIC trial, which is in line with findings in the PACIFIC-R multinational observational trial where the median time to treatment was 52 days (survival not characterized).^29^ Our results suggest there may be flexibility in the time to initiate immunotherapy after the completion of radiation. More specifically, the PACIFIC trial dictated that immunotherapy should be initiated within 6 weeks of the completion of radiation. This timeframe was chosen because of the perception that the initiation of immunotherapy is time sensitive, and a number of contemporary trials have also included similar time-to-initiation restrictions.^30,31,32^ In fact, an unplanned subset analysis of the PACIFIC trial suggested that those who started within 2 weeks of completing radiation had superior outcomes compared with those who started between 2 and 6 weeks; however, our findings did not identify a significant advantage of starting immunotherapy earlier (eTable 7 in the Supplement).^33^

PACIFIC trial treatment recommendations are unlikely to be fully complied with by all patients in a clinical setting. Some patients struggle with chemoradiation for stage III NSCLC (grade 3 and 4 adverse event rates in the chemoradiation arm of the PACIFIC trial were 26.1%),^5,34,35^ and therefore a percentage of patients may require additional time to recover from chemoradiation prior to starting immunotherapy. In the current study, findings would suggest a delay in initiation beyond 6 weeks is not necessarily a contraindication to immunotherapy. The radiation dose was variable in the current population and the advantage of immunotherapy was less clear in patients treated outside the recommended range. This subset (221 patients) may have been underpowered. Among patients treated with immunotherapy, we did not identify a superior radiation dose (eTable 5 in the Supplement). However, prior studies of nonsurgically managed stage III NSCLC (chemoradiation alone) have tended to favor radiation doses of at least 60 Gy.^36^

### Limitations

This study had several limitations in addition to those traditionally associated with observational research. Although this was a population study, it is possible that some of the patients were participants in clinical trials (eg, we estimate around 150 PACIFIC trial patients were from US hospitals, some of whom were likely captured by the NCDB), which would make our sample less reflective of clinical practice. Only 1.0% of patients receiving immunotherapy were indicated as clinical trial participants per the NCDB data variable, but we suspect this variable to have had low sensitivity. The observation that the majority of patients were treated differently than the PACIFIC trial protocol would support the conclusion that most of our study group were not a part of the trial. The NCDB does not capture the type of immunotherapy used (eg, anti–PD-L1, programmed cell death 1, cytotoxic T-lymphocyte associated antigen-4 immunoglobulin, etc) and the immunotherapy variable includes some agents that are not checkpoint inhibitors such as bevacizumab. However, during the timeframe studied, the vast majority of immunotherapy would have been directed at the programmed cell death 1 axis. It is possible that the magnitude of benefit of immunotherapy could vary by the type of checkpoint inhibitor that was used. The NCDB does not capture a number of important factors involved in immunotherapy response (eg, PD-L1, estimated glomerular filtration rate, smoking status), which could influence results and the applicability of the findings. However, the PACIFIC trial itself was masked to PD-L1 and estimated glomerular filtration rate status, and it has been documented that the use of PD-L1 status to direct therapy is not uniform across practitioners.^5,37^ Similarly, the NCDB does not capture chemotherapy agent and whether chemotherapy was given concurrently with radiation. However, it is likely many patients received platinum-containing chemotherapy and concurrent chemoradiation given the associated survival advantage and class IA guideline recommendations.^38,39^ The NCDB also does not capture progression free survival, which was a main outcome in the PACIFIC trial; however, overall survival is still an important metric and was another main outcome measured in the PACIFIC trial. It is also worth noting that a tumor’s designation as being unresectable is to some degree subjective and likely varies from hospital to hospital and across clinical teams. We do believe one strength of observational research in large hospital data sets is the incorporation of data across highly variable environments, which is likely more reflective of clinical practice.

The NCDB does not track safety data for treatment. Because the immunotherapy cohort experienced superior overall survival, it appears that immunotherapy was associated with a survival advantage in spite of any treatment related adversity recognizing that patient goals of care extend beyond overall survival.

Lastly, there is no way of knowing how patients were selected to receive immunotherapy after chemotherapy and radiation including response to treatment, which could introduce selection bias. The NCDB captures clinical trial participation; however, the fidelity of this variable is low. In the years before the PACIFIC trial results were widely known in 2017, patients seeking early access to innovative treatment could be motivated by poor prognostic attributes through compassionate use. However, it is also possible the immunotherapy cohort had favorable tumor attributes not captured by NCDB, which would make immunotherapy appear more effective.

## Conclusions

Overall, the results of this retrospective cohort study suggest that the benefit of immunotherapy after chemoradiation for stage III NSCLC may extend to the general population in the US, including patients who are older and those who are socioeconomically disadvantaged. There may be flexibility in the multimodality treatment planning, including the time to initiate immunotherapy, which may alleviate barriers to immunotherapy treatment in some patients. Further study to refine the implications of immunotherapy in clinical stage III NSCLC in a clinical setting is justified.

## Notes

Supplement. **eTable 1.** Propensity-Matched Baseline Characteristics and Standardized Mean Differences of Patients Receiving Chemotherapy and Radiation Followed By Immunotherapy vs Chemotherapy and Radiation Only **eTable 2.** CONSORT Diagram **eFigure.** Unmatched Kaplan-Meier Curve with Survival of Stage III NSCLC Patients Who Received Chemotherapy and Radiation Only Compared to Patients Who Received Chemotherapy and Radiation Followed by Immunotherapy **eTable 3.** Cox Proportional Hazards Model of Stage III NSCLC Patients Who Received Chemotherapy and Radiation With Immunotherapy vs Chemotherapy and Radiation Only **eTable 4.** Cox Proportional Hazards Model of Patients Who Received Chemotherapy and Radiation With Immunotherapy vs Patients Who Received Chemotherapy and Radiation Only for Stage III NSCLC With Radiation Doses as Covariates **eTable 5.** Cox Proportional Hazards Model of Patients Who Received Chemotherapy and Radiation With Immunotherapy for Stage III NSCLC With Radiation Dose as a Covariate **eTable 6.** Cox Proportional Hazards Model of Patients Who Received Chemotherapy and Radiation With Immunotherapy vs Patients Who Received Chemotherapy and Radiation Only for Stage III NSCLC With Time Between Radiation Completion and Starting Immunotherapy as a Covariate **eTable 7.** Cox Proportional Hazards Model of Patients Who Received Chemotherapy and Radiation With Immunotherapy for Stage III NSCLC With Time to Immunotherapy Within the PACIFIC Trial Limits and Outside the PACIFIC Trial Limits as a Covariate. **eTable 8.** Previously Reported Hazard Ratios for Overall Survival Comparing Durvalumab Plus Chemoradiation to Chemoradiation Alone **eTable 9.** *P* Values Before and After Benjamini-Hochberg Multiple Testing Adjustment

## References

[1] American Cancer Society. Cancer Facts and Figures. Published 2021. Accessed January 3, 2022. https://www.cancer.org/research/cancer-facts-statistics/all-cancer-facts-figures/cancer-facts-figures-2021.html

[2] Ganti AK, Klein AB, Cotarla I, Seal B, Chou E. Update of incidence, prevalence, survival, and initial treatment in patients with non–small cell lung cancer in the US. JAMA Oncol. 2021;7(12):1824-1832. doi:10.1001/jamaoncol.2021.4932 34673888 PMC8532041

[3] Spigel DR, Faivre-Finn C, Gray JE, . Five-year survival outcomes with durvalumab after chemoradiotherapy in unresectable stage III NSCLC: an update from the PACIFIC trial. J Clin Oncol. 2021;39(15_suppl):8511-8511. doi:10.1200/JCO.2021.39.15_suppl.8511

[4] Antonia SJ, Villegas A, Daniel D, ; PACIFIC Investigators. Overall survival with durvalumab after chemoradiotherapy in stage III NSCLC. N Engl J Med. 2018;379(24):2342-2350. doi:10.1056/NEJMoa1809697 30280658

[5] Antonia SJ, Villegas A, Daniel D, ; PACIFIC Investigators. Durvalumab after chemoradiotherapy in stage III non–small-cell lung cancer. N Engl J Med. 2017;377(20):1919-1929. doi:10.1056/NEJMoa1709937 28885881

[6] Faivre-Finn C, Vicente D, Kurata T, . Four-year survival with durvalumab after chemoradiotherapy in stage III NSCLC—an update from the PACIFIC trial. J Thorac Oncol. 2021;16(5):860-867. doi:10.1016/j.jtho.2020.12.015 33476803

[7] US Food and Drug Administration. FDA Approves Durvalumab after Chemoradiation for Unresectable Stage III NSCLC. Press release. Updated February 20, 2018. Accessed May 2, 2022. https://www.fda.gov/drugs/resources-information-approved-drugs/fda-approves-durvalumab-after-chemoradiation-unresectable-stage-iii-nsclc

[8] National Comprehensive Cancer Network. Non–small Cell Lung Cancer Guidelines (Version 1.2022). Published 2022. Accessed January 20, 2022. https://www.nccn.org/guidelines/guidelines-detail?category=1&id=1450

[9] Averitt AJ, Weng C, Ryan P, Perotte A. Translating evidence into practice: eligibility criteria fail to eliminate clinically significant differences between real-world and study populations. NPJ Digit Med. 2020;3:67. doi:10.1038/s41746-020-0277-8 32411828 PMC7214444

[10] Fehrenbacher L, Ackerson L, Somkin C. Randomized clinical trial eligibility rates for chemotherapy (CT) and antiangiogenic therapy (AAT) in a population-based cohort of newly diagnosed non–small cell lung cancer (NSCLC) patients. J Clin Oncol. 2009;27(15_suppl):6538-6538. doi:10.1200/jco.2009.27.15_suppl.6538

[11] Howe LD, Tilling K, Galobardes B, Lawlor DA. Loss to follow-up in cohort studies: bias in estimates of socioeconomic inequalities. Epidemiology. 2013;24(1):1-9. doi:10.1097/EDE.0b013e31827623b1 23211345 PMC5102324

[12] Martinez PL, McGarrity LA, Turner NA, . Self-pay payer status predicts long-term loss to follow-up after bariatric surgery. Obes Surg. 2021;31(4):1590-1596. doi:10.1007/s11695-020-05161-4 33515181

[13] Vallabhajosyula S, Kumar V, Sundaragiri PR, . Influence of primary payer status on the management and outcomes of ST-segment elevation myocardial infarction in the United States. PLoS One. 2020;15(12):e0243810. doi:10.1371/journal.pone.0243810 33338071 PMC7748387

[14] Ryan KJ, Skinner KE, Fernandes AW, . Real-world treatment patterns among patients with unresected stage III non–small-cell lung cancer. Future Oncol. 2019;15(25):2943-2953. doi:10.2217/fon-2018-0939 31037966

[15] Abdel-Rahman O, Koski S, Mulder K. Real-world patterns of chemotherapy administration and attrition among patients with metastatic colorectal cancer. Int J Colorectal Dis. 2021;36(3):493-499. doi:10.1007/s00384-020-03778-6 33068162

[16] American College of Surgeons. About the National Cancer Database. ACS website. Accessed February 3, 2022. https://www.facs.org/quality-programs/cancer/ncdb/about

[17] Boffa DJ, Rosen JE, Mallin K, . Using the National Cancer Database for outcomes research: a review. JAMA Oncol. 2017;3(12):1722-1728. doi:10.1001/jamaoncol.2016.6905 28241198

[18] National Cancer Database Participant User File. 2018 Data Dictionary. Updated October 2018. Accessed February 8, 2022. https://www.facs.org/-/media/files/quality-programs/cancer/ncdb/puf_data_dictionary.ashx

[19] Sterne JAC, White IR, Carlin JB, . Multiple imputation for missing data in epidemiological and clinical research: potential and pitfalls. BMJ. 2009;338:b2393. doi:10.1136/bmj.b2393 19564179 PMC2714692

[20] Rubin DB. Multiple Imputations for Nonresponse in Surveys. John Wiley & Sons, Inc; 1987. doi:10.1002/9780470316696

[21] Yadav K, Lewis RJ. Immortal time bias in observational studies. JAMA. 2021;325(7):686-687. doi:10.1001/jama.2020.9151 33591334

[22] Benjamini Y, Hochberg Y. Controlling the false discovery rate: a practical and powerful approach to multiple testing. J R Stat Soc B. 1995;57(1):289-300. doi:10.1111/j.2517-6161.1995.tb02031.x

[23] Desilets A, Blanc-Durand F, Lau S, . Durvalumab therapy following chemoradiation compared with a historical cohort treated with chemoradiation alone in patients with stage III non–small cell lung cancer: a real-world multicentre study. Eur J Cancer. 2021;142:83-91. doi:10.1016/j.ejca.2020.10.008 33242835

[24] Fukui T, Hosotani S, Soda I, . Current status and progress of concurrent chemoradiotherapy in patients with locally advanced non–small cell lung cancer prior to the approval of durvalumab. Thorac Cancer. 2020;11(4):1005-1014. doi:10.1111/1759-7714.13357 32057187 PMC7113036

[25] Sankar K, Bryant AK, Strohbehn GW, . Real world outcomes versus clinical trial results of durvalumab maintenance in veterans with stage III non–small cell lung cancer. Cancers (Basel). 2022;14(3):614. doi:10.3390/cancers14030614 35158881 PMC8833364

[26] Socinski MA, Özgüroğlu M, Villegas A, . Durvalumab after concurrent chemoradiotherapy in elderly patients with unresectable stage III non–small-cell lung cancer (PACIFIC). Clin Lung Cancer. 2021;22(6):549-561. doi:10.1016/j.cllc.2021.05.009 34294595

[27] Ermer T, Walters SL, Canavan ME, . Understanding the implications of Medicaid expansion for cancer care in the US: a review. JAMA Oncol. 2022;8(1):139-148. doi:10.1001/jamaoncol.2021.4323 34762101

[28] Johnson T, Bandini LAM, Martin K, . The state of cancer care in America: impact of state policy on access to high-quality cancer care. J Natl Compr Canc Netw. 2020;18(4):400-404. doi:10.6004/jnccn.2020.7541 32259786

[29] McDonald F, Mornex F, Garassino MC, . 79MO PACIFIC-R: real-world characteristics of unresectable stage III NSCLC patients treated with durvalumab after chemoradiotherapy. J Thorac Oncol. 2021;16(4):S738-S739. doi:10.1016/S1556-0864(21)01921-3

[30] A study of atezolizumab and tiragolumab compared with durvalumab in participants with locally advanced, unresectable stage III non–small cell lung cancer (NSCLC) (SKYSCRAPER-03). ClinicalTrials.gov identifier: NCT04513925. Updated June 15, 2022. Accessed February 16, 2022. https://clinicaltrials.gov/ct2/show/NCT04513925

[31] A study to determine safety of durvalumab after sequential chemo radiation in patients with unresectable stage III non–small cell lung cancer. ClinicalTrials.gov identifier: NCT03693300. Updated May 9, 2022. Accessed February 16, 2022. https://clinicaltrials.gov/ct2/show/NCT03693300

[32] Durvalumab followed by chemoradiation and consolidation durvalumab for stage III non–small cell lung cancer. ClinicalTrials.gov identifier: NCT04364048. Updated January 21, 2022. Accessed February 16, 2022. https://clinicaltrials.gov/ct2/show/NCT04364048

[33] Faivre-Finn C, Spigel DR, Senan S, . Efficacy and safety evaluation based on time from completion of radiotherapy to randomization with durvalumab or placebo in pts from PACIFIC. Ann Oncol. 2018;29(8_suppl):VIII488. doi:10.1093/annonc/mdy291

[34] Bradley JD, Hu C, Komaki RR, . Long-term results of NRG Oncology RTOG 0617: standard- versus high-dose chemoradiotherapy with or without cetuximab for unresectable stage III non–small-cell lung cancer. J Clin Oncol. 2020;38(7):706-714. doi:10.1200/JCO.19.01162 31841363 PMC7048161

[35] LeClair JN, Merl MY, Cohenuram M, Luon D. Real-world incidence of pneumonitis in patients receiving durvalumab. Clin Lung Cancer. 2021;23(1):34-42. doi:10.1016/j.cllc.2021.08.006 34556401

[36] Saffarzadeh AG, Canavan M, Resio BJ, . Optimal radiation dose for stage III lung cancer—should “definitive” radiation doses be used in the preoperative setting? JTO Clin Res Rep. 2021;2(8):100201. doi:10.1016/j.jtocrr.2021.100201 34590044 PMC8474436

[37] Leapman MS, Presley CJ, Zhu W, . Association of programmed cell death ligand 1 expression status with receipt of immune checkpoint inhibitors in patients with advanced non–small cell lung cancer. JAMA Netw Open. 2020;3(6):e207205. doi:10.1001/jamanetworkopen.2020.7205 32511721 PMC7280954

[38] Detterbeck FC, Lewis SZ, Diekemper R, Addrizzo-Harris D, Alberts WM. Executive summary: diagnosis and management of lung cancer, 3rd ed: American College of Chest Physicians evidence-based clinical practice guidelines. Chest. 2013;143(5)(suppl):7S-37S. doi:10.1378/chest.12-2377 23649434

[39] Curran WJ Jr, Paulus R, Langer CJ, . Sequential vs. concurrent chemoradiation for stage III non–small cell lung cancer: randomized phase III trial RTOG 9410. J Natl Cancer Inst. 2011;103(19):1452-1460. doi:10.1093/jnci/djr325 21903745 PMC3186782

