# Supplementary material for: Immunotherapy After Chemotherapy and Radiation for Clinical Stage III Lung Cancer
Source: JAMA Netw Open. 2022 Aug 4;5(8):e2224478. doi: 10.1001/jamanetworkopen.2022.24478 (PMC9353596; doi:10.1001/jamanetworkopen.2022.24478)
Supplement: Supplement. — eTable 1. Propensity-Matched Baseline Characteristics and Standardized Mean Differences of Patients Receiving Chemotherapy and Radiation Followed By Immunotherapy vs Chemotherapy and Radiation Only eTable 2. CONSORT Diagram eFigure. Unmatched Kaplan-Meier Curve with Survival of Stage III NSCLC Patients Who Received Chemotherapy and Radiation Only Compared to Patients Who Received Chemotherapy and Radiation Followed by Immunotherapy eTable 3. Cox Proportional Hazards Model of Stage III NSCLC Patients Who Received Chemotherapy and Radiation With Immunotherapy vs Chemotherapy and Radiation Only eTable 4. Cox Proportional Hazards Model of Patients Who Received Chemotherapy and Radiation With Immunotherapy vs Patients Who Received Chemotherapy and Radiation Only for Stage III NSCLC With Radiation Doses as Covariates eTable 5. Cox Proportional Hazards Model of Patients Who Received Chemotherapy and Radiation With Immunotherapy for Stage III NSCLC With Radiation Dose as a Covariate eTable 6. Cox Proportional Hazards Model of Patients Who Received Chemotherapy and Radiation With Immunotherapy vs Patients Who Received Chemotherapy and Radiation Only for Stage III NSCLC With Time Between Radiation Completion and Starting Immunotherapy as a Covariate eTable 7. Cox Proportional Hazards Model of Patients Who Received Chemotherapy and Radiation With Immunotherapy for Stage III NSCLC With Time to Immunotherapy Within the PACIFIC Trial Limits and Outside the PACIFIC Trial Limits as a Covariate. eTable 8. Previously Reported Hazard Ratios for Overall Survival Comparing Durvalumab Plus Chemoradiation to Chemoradiation Alone eTable 9. P Values Before and After Benjamini-Hochberg Multiple Testing Adjustment [file jamanetwopen-e2224478-s001.pdf]

Supplemental Online Content

Pichert MD, Canavan ME, Maduka RC, et al. Immunotherapy after chemotherapy and radiation for clinical stage III lung cancer. *JAMA Netw Open.* 2022;5(8):e2224478. doi:10.1001/jamanetworkopen.2022.24478

eTable 1. Propensity-Matched Baseline Characteristics and Standardized Mean Differences of Patients Receiving Chemotherapy and Radiation Followed By Immunotherapy vs Chemotherapy and Radiation Only

eTable 2. CONSORT Diagram

eFigure. Unmatched Kaplan-Meier Curve with Survival of Stage III NSCLC Patients Who Received Chemotherapy and Radiation Only Compared to Patients Who Received Chemotherapy and Radiation Followed by Immunotherapy

eTable 3. Cox Proportional Hazards Model of Stage III NSCLC Patients Who Received Chemotherapy and Radiation With Immunotherapy vs Chemotherapy and Radiation Only

eTable 4. Cox Proportional Hazards Model of Patients Who Received Chemotherapy and Radiation With Immunotherapy vs Patients Who Received Chemotherapy and Radiation Only for Stage III NSCLC With Radiation Doses as Covariates

eTable 5. Cox Proportional Hazards Model of Patients Who Received Chemotherapy and Radiation With Immunotherapy for Stage III NSCLC With Radiation Dose as a Covariate

eTable 6. Cox Proportional Hazards Model of Patients Who Received Chemotherapy and Radiation With Immunotherapy vs Patients Who Received Chemotherapy and Radiation Only for Stage III NSCLC With Time Between Radiation Completion and Starting Immunotherapy as a Covariate

eTable 7. Cox Proportional Hazards Model of Patients Who Received Chemotherapy and Radiation With Immunotherapy for Stage III NSCLC With Time to Immunotherapy Within the PACIFIC Trial Limits and Outside the PACIFIC Trial Limits as a Covariate

eTable 8. Previously Reported Hazard Ratios for Overall Survival Comparing Durvalumab Plus Chemoradiation to Chemoradiation Alone

eTable 9. *P* Values Before and After Benjamini-Hochberg Multiple Testing Adjustment

This supplemental material has been provided by the authors to give readers additional information about their work.

© 2022 Pichert MD et al. *JAMA Network Open.*

eTable 1. Propensity-Matched Baseline Characteristics and Standardized Mean Differences of Patients Receiving Chemotherapy and Radiation Followed By Immunotherapy vs Chemotherapy and Radiation Only.

	**Chemoradiation Only (%)**	**Chemoradiation Followed by Immunotherapy (%)**	**Standardized Mean Difference**	**P value**
Total Patients	2,594 (66.7)	1,297 (33.3)		
Age (yrs)				.76
20-49	98 (64.1)	55 (36)	Ref	
50-64	1,016 (66.2)	519 (33.8)	-0.01734	
65-74	1,058 (67.5)	509 (32.5)	0.03148	
≥75	422 (66.4)	214 (33.7)	-0.00625	
Sex				.73
Male	1,381 (66.4)	698 (33.6)	Ref	
Female	1,213 (66.9)	599 (33.1)	0.01159	
Race				.69
White	2,193 (66.7)	1,094 (33.3)	Ref	
Black	309 (67)	152 (33)	0.00597	
Asian	72 (66.7)	36 (33.3)	0.0	
Other/Unknown	20 (57.1)	15 (42.9)	-0.03947	
Ethnicity				.59
Non-Hispanic	2,505 (66.8)	1,245 (33.2)	Ref	
Hispanic	46 (61.3)	29 (38.7)	-0.03301	
Unknown	43 (65.2)	23 (34.9)	-0.00891	
Insurance				.70
Private	844 (67.1)	413 (32.9)	Ref	
Medicare	1,394 (66.9)	689 (33.1)	0.01237	
Medicaid	212 (63.1)	124 (36.9)	-0.04884	
Uninsured	50 (63.3)	29 (36.7)	-0.0216	
Other Government	58 (68.2)	27 (31.8)	0.01061	
Unknown	36 (70.6)	15 (29.4)	0.02064	
Median Income				.86
<$38,000	401 (66.1)	206 (33.9)	Ref	
$38,000-$47,999	596 (67.7)	285 (32.4)	0.02401	
≥$48,000	1,174 (66.2)	600 (33.8)	-0.02012	
Unknown	423 (67.3)	206 (32.8)	0.01154	
Year of Diagnosis				.89
2015	125 (65.5)	66 (34.6)	Ref	
2016	163 (67.6)	78 (32.4)	0.01123	
2017	2,306 (66.7)	1,153 (33.3)	0.0	
Charlson-Deyo Score				1.00
0	1,501 (66.7)	748 (33.3)	Ref	
1	678 (66.6)	340 (33.4)	-0.00175	
2	259 (66.4)	131 (33.6)	-0.00385	
3	156 (66.7)	78 (33.3)	0.0	

eTable 1 Continued.

Histology				.84
Adenocarcinoma	1,207 (67.1)	591 (32.9)	Ref	
Squamous Cell	1,144 (66.3)	581 (33.7)	-0.01396	
Large Cell	25 (71.4)	10 (28.6)	0.02079	
Other	218 (65.5)	115 (34.5)	-0.01647	
T Category				.25
1	464 (67.5)	223 (32.5)	Ref	
2	757 (66.9)	374 (33.1)	0.00765	
3	618 (65.4)	327 (34.6)	-0.03226	
4	712 (66.5)	359 (33.5)	-0.00518	
Unknown	32 (69.6)	14 (30.4)	0.01442	
N Category				.68
0	167 (66)	86 (34)	Ref	
1	158 (62.5)	95 (37.6)	-0.04933	
2	1,590 (67.1)	780 (32.9)	0.02368	
3	675 (66.9)	334 (33.1)	0.00616	
Unknown	4 (66.7)	2 (33.3)	0.0	
Facility Type				1.00
Non-Academic	1,666 (66.6)	834 (33.4)	-0.00161	
Academic	918 (66.7)	458 (33.3)	Ref	
Unknown	10 (66.7)	5 (33.3)	0.0	
Region				.65
Northeast	493 (66)	254 (34)	Ref	
Midwest	899 (68.3)	417 (31.7)	0.05315	
South	903 (65.9)	468 (34.1)	-0.0266	
West	289 (65.4)	153 (34.6)	-0.02057	

© 2022 Pichert MD et al. *JAMA Network Open.*

eTable 2. Consort Diagram.

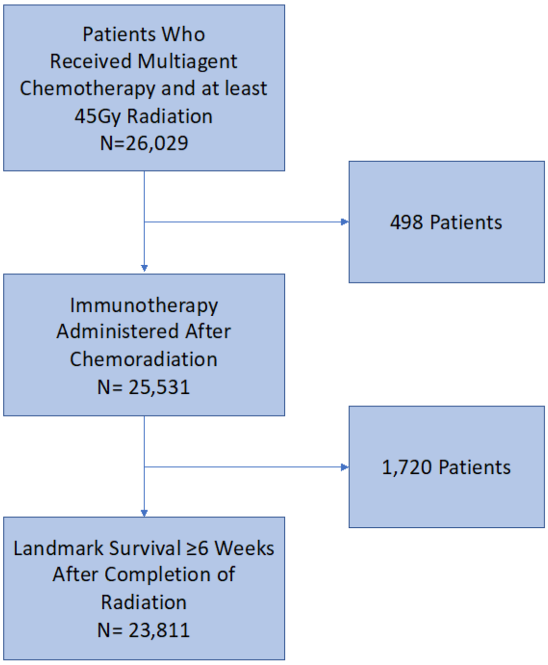

© 2022 Pichert MD et al. *JAMA Network Open.*

eFigure 1. Unmatched Kaplan-Meier Curve with Survival of Stage III NSCLC Patients who Received Chemotherapy and Radiation Only Compared to Patients who Received Chemotherapy and Radiation Followed by Immunotherapy.

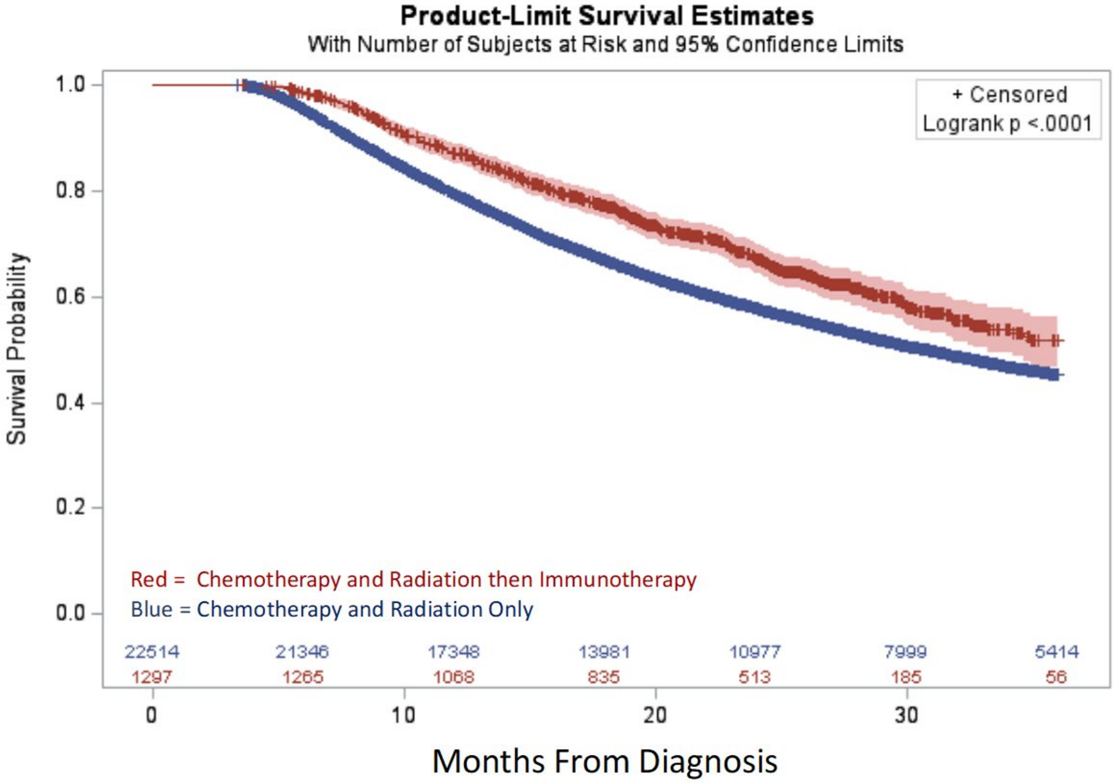

[a]Chemotherapy and Radiation only median survival is 30.72 (29.90, 31.41)
[b]Immunotherapy median survival is 36.21 (33.22, 48.82)

eTable 3. Cox Proportional Hazards Model of Stage III NSCLC Patients who Received Chemotherapy and Radiation with Immunotherapy vs Chemotherapy and Radiation Only.

	**Hazard Ratio (95% CI)**	**P value**
Immunotherapy		
Chemoradiation Only	Reference	
Immunotherapy After Chemoradiation	0.74 (0.67 - 0.82)	<.001
Age (yrs)		
20-49	Reference	
50-64	1.13 (1.01 - 1.27)	.03
65-74	1.22 (1.09 - 1.38)	.001
≥75	1.51 (1.33 - 1.71)	<.001
Sex		
Male	Reference	
Female	0.82 (0.79 - 0.85)	<.001
Race		
White	Reference	
Black	0.87 (0.82 - 0.92)	<.001
Asian	0.70 (0.61 - 0.8)	<.001
Ethnicity		
Non-Hispanic	Reference	
Hispanic	0.79 (0.70 - 0.89)	<.001
Insurance		
Private	Reference	
Medicare	1.15 (1.09 - 1.21)	<.001
Medicaid	1.16 (1.08 - 1.24)	<.001
Uninsured	1.12 (0.97 - 1.28)	.12
Other Government	1.03 (0.90 - 1.17)	.70
Median Income		
<$38,000	Reference	
$38,000-$47,999	0.98 (0.92 - 1.04)	.45
≥$48,000	0.92 (0.87 - 0.97)	.002
Year of Diagnosis		
2015	Reference	
2016	0.93 (0.89 - 0.97)	<.001
2017	0.92 (0.88 - 0.97)	.001
Charlson-Deyo Score		
0	Reference	
1	1.09 (1.05 - 1.14)	<.001
2	1.17 (1.1 - 1.24)	<.001
3	1.29 (1.19 - 1.4)	<.001
Histology		
Adenocarcinoma	Reference	
Squamous Cell	1.21 (1.16 - 1.26)	<.001
Large Cell	1.41 (1.22 - 1.64)	<.001
Other	1.23 (1.16 - 1.31)	<.001

© 2022 Pichert MD et al. *JAMA Network Open.*

eTable 3 Continued.

T Category		
0	0.85 (0.62 - 1.17)	.31
1	Reference	
2	1.22 (1.16 - 1.29)	<.001
3	1.39 (1.31 - 1.47)	<.001
4	1.45 (1.37 - 1.54)	<.001
N Category		
0	Reference	
1	1.10 (1.00 - 1.22)	.05
2	1.36 (1.26 - 1.47)	<.001
3	1.66 (1.53 - 1.81)	<.001
Facility Type		
Non-Academic	Reference	
Academic	0.88 (0.84 - 0.92)	<.001
Region		
Northeast	Reference	
Midwest	1.00 (0.94 - 1.06)	1.00
South	0.96 (0.91 - 1.02)	.21
West	0.94 (0.87 - 1.02)	.14

[a]Age was run as a categorical variable, as there are differences in treatment safety and effectiveness outcomes associated with specific subsets of older patients. Separate model using age a continuous variable was performed and results did not differ (data available upon request).

[b]Total N = 23,811 with 12,481 events

© 2022 Pichert MD et al. *JAMA Network Open.*

eTable 4. Cox Proportional Hazards Model of Patients who Received Chemotherapy and Radiation with Immunotherapy vs Patients who Received Chemotherapy and Radiation Only for Stage III NSCLC With Radiation Doses as Covariates.

	**Hazard Ratio (95% CI)**	**P value**
Radiation Dose		
Less Than PACIFIC (45-53 Gy)	0.97 (0.93 – 1.03)	.34
PACIFIC Low (54-50 Gy)	1.01 (0.94 – 1.08)	.84
PACIFIC Mid (60 Gy)	Reference	
PACIFIC High (61-66 Gy)	0.99 (0.95 – 1.04)	.79
Greater Than PACIFIC (≥67 Gy)	0.97 (0.89 – 1.06)	.53
Immunotherapy		
Chemoradiation Only	Reference	
Immunotherapy After Chemoradiation	0.74 (0.67 - 0.82)	<.001
Age (yrs)		
20-49	Reference	
50-64	1.13 (1.01 - 1.27)	.03
65-74	1.22 (1.08 - 1.38)	.001
≥75	1.51 (1.33 - 1.70)	<.001
Sex		
Male	Reference	
Female	0.82 (0.79 - 0.85)	<.001
Race		
White	Reference	
Black	0.87 (0.82 - 0.92)	<.001
Asian	0.70 (0.61 - 0.80)	<.001
Ethnicity		
Non-Hispanic	Reference	
Hispanic	0.79 (0.70 - 0.89)	<.001
Insurance		
Private	Reference	
Medicare	1.15 (1.09 - 1.21)	<.001
Medicaid	1.16 (1.08 - 1.24)	<.001
Uninsured	1.11 (0.97 - 1.28)	.12
Other Government	1.02 (0.90 - 1.17)	.71
Median Income		
<$38,000	Reference	
$38,000-$47,999	0.98 (0.92 - 1.04)	.44
≥$48,000	0.92 (0.87 - 0.97)	.002
Year of Diagnosis		
2015	Reference	
2016	0.93 (0.89 - 0.97)	<.001
2017	0.92 (0.88 - 0.97)	<.001

© 2022 Pichert MD et al. *JAMA Network Open.*

eTable 4 Continued.

Charlson-Deyo Score		
0	Reference	
1	1.09 (1.05 - 1.14)	<.001
2	1.17 (1.1 - 1.24)	<.001
3	1.29 (1.19 - 1.4)	<.001
Histology		
Adenocarcinoma	Reference	
Squamous Cell	1.21 (1.16 - 1.26)	<.001
Large Cell	1.41 (1.22 - 1.64)	<.001
Other	1.23 (1.15 - 1.31)	<.001
T Category		
0	0.85 (0.62 - 1.17)	.31
1	Reference	
2	1.22 (1.16 - 1.29)	<.001
3	1.39 (1.31 - 1.47)	<.001
4	1.45 (1.37 - 1.54)	<.001
N Category		
0	Reference	
1	1.10 (1.00 - 1.22)	.05
2	1.36 (1.26 - 1.47)	<.001
3	1.66 (1.52 - 1.81)	<.001
Facility Type		
Non-Academic	Reference	
Academic	0.88 (0.84 - 0.92)	<.001
Region		
Northeast	Reference	
Midwest	1.00 (0.94 - 1.06)	.99
South	0.96 (0.91 - 1.02)	.21
West	0.94 (0.87 - 1.02)	.14

[a]Total N = 23,811 with 12,481 events

© 2022 Pichert MD et al. *JAMA Network Open.*

eTable 5. Cox Proportional Hazards Model of Patients who Received Chemotherapy and Radiation with Immunotherapy for Stage III NSCLC with Radiation Dose as a Covariate.

	**Hazard Ration (95% CI)**	**P Value**
Radiation Dose		
40-53 Gy	1.16 (0.87 - 1.55)	.32
54-59 Gy	1.00 (0.69 - 1.46)	.99
60 Gy	Reference	
61-66 GY	0.94 (0.74 - 1.20)	.64
≥67GY	0.92 (0.57 - 1.48)	.73
Age (yrs)		
20-49	Reference	
50-64	1.13 (0.66 - 1.94)	.66
65-74	0.94 (0.53 - 1.69)	.85
≥75	0.98 (0.53 - 1.82)	.96
Sex		
Male	Reference	
Female	0.86 (0.71 - 1.05)	.13
Race		
White	Reference	
Black	0.75 (0.54 - 1.05)	.09
Asian	0.28 (0.10 - 0.76)	.01
Ethnicity		
Non-Hispanic	Reference	
Hispanic	0.74 (0.35 - 1.57)	.44
Insurance		
Private	Reference	
Medicare	1.11 (0.84 - 1.47)	.45
Medicaid	0.95 (0.65 - 1.37)	.77
Uninsured	1.09 (0.55 - 2.16)	.80
Other Government	0.78 (0.38 - 1.57)	.48
Median Income		
<$38,000	Reference	
$38,000-$47,999	0.84 (0.60 - 1.17)	.29
≥$48,000	0.72 (0.53 - 0.97)	.03

© 2022 Pichert MD et al. *JAMA Network Open.*

**eTable 5 Continued.**<br>Year of Diagnosis		
2015	Reference	
2016	1.00 (0.63 - 1.58)	.99
2017	0.73 (0.51 - 1.06)	.09
Charlson-Deyo Score		
0	Reference	
1	1.14 (0.91 - 1.43)	.24
2	1.47 (1.07 - 2.01)	.02
3	1.50 (1.03 - 2.18)	.04
Histology		
Adenocarcinoma	Reference	
Squamous Cell	1.54 (1.25 - 1.89)	<.001
Large Cell	2.13 (0.90 - 5.04)	.09
Other	0.77 (0.51 - 1.17)	.23
T Category		
1	Reference	
2	1.34 (0.98 - 1.82)	.06
3	1.46 (1.05 - 2.02)	.02
4	1.51 (1.08 - 2.11)	.02
N Category		
0	Reference	
1	0.87 (0.49 - 1.53)	.62
2	1.21 (0.78 - 1.86)	.39
3	1.66 (1.04 - 2.64)	.03
Facility Type		
Non-Academic	Reference	
Academic	0.80 (0.64 - 0.99)	.04
Region		
Northeast	Reference	
Midwest	0.97 (0.73 - 1.29)	.83
South	0.96 (0.72 - 1.27)	.77
West	0.85 (0.58 - 1.24)	.39

[a]Total N = 1,297 with 439 events

© 2022 Pichert MD et al. *JAMA Network Open.*

eTable 6. Cox Proportional Hazards Model of Patients who Received Chemotherapy and Radiation with Immunotherapy vs Patients who Received Chemotherapy and Radiation Only for Stage III NSCLC with Time Between Radiation Completion and Starting Immunotherapy as a Covariate.

	**Hazard Ratio (95% CI)**	**P value**
Time to Immunotherapy		
No Immunotherapy	Reference	
≤6 Weeks	0.78 (0.68 - 0.90)	<.001
7-9 Weeks	0.71 (0.59 - 0.84)	<.001
10-12 Weeks	0.61 (0.47 - 0.79)	<.001
>12 Weeks	0.75 (0.61 - 0.92)	.01
Age (yrs)		
20-49	Reference	
50-64	1.13 (1.01 - 1.27)	.03
65-74	1.22 (1.09 - 1.38)	.01
≥75	1.51 (1.33 - 1.71)	<.001
Sex		
Male	Reference	
Female	0.82 (0.79 - 0.85)	<.001
Race		
White	Reference	
Black	0.87 (0.82 - 0.92)	<.001
Asian	0.70 (0.61 - 0.80)	<.001
Ethnicity		
Non-Hispanic	Reference	
Hispanic	0.79 (0.70 - 0.89)	<.001
Insurance		
Private	Reference	
Medicare	1.15 (1.09 - 1.21)	<.001
Medicaid	1.15 (1.07 - 1.24)	<.001
Uninsured	1.12 (0.98 - 1.28)	.11
Other Government	1.02 (0.90 - 1.17)	.71
Median Income		
<$38,000	Reference	
$38,000-$47,999	0.98 (0.92 - 1.04)	.45
≥$48,000	0.92 (0.87 - 0.97)	.002
Year of Diagnosis		
2015	Reference	
2016	0.93 (0.89 - 0.97)	.001
2017	0.92 (0.88 - 0.97)	.001
Charlson-Deyo Score		
0	Reference	
1	1.09 (1.05 - 1.14)	<.001
2	1.17 (1.10 - 1.24)	<.001
3	1.29 (1.19 - 1.40)	<.001
Histology		
Adenocarcinoma	Reference	
Squamous Cell	1.21 (1.16 - 1.26)	<.001
Large Cell	1.41 (1.22 - 1.63)	<.001
Other	1.23 (1.16 - 1.31)	<.001

© 2022 Pichert MD et al. *JAMA Network Open.*

eTable 6 Continued.

T Category		
0	0.85 (0.62 - 1.17)	.32
1	Reference	
2	1.22 (1.16 - 1.29)	<.001
3	1.39 (1.32 - 1.47)	<.001
4	1.45 (1.37 - 1.54)	<.001
N Category		
0	Reference	
1	1.10 (1 - 1.22)	.06
2	1.36 (1.26 - 1.47)	<.001
3	1.66 (1.53 - 1.81)	<.001
Facility Type		
Non-Academic	Reference	
Academic	0.88 (0.84 - 0.92)	<.001
Region		
Northeast	Reference	
Midwest	1.00 (0.94 - 1.06)	.99
South	0.96 (0.91 - 1.02)	.20
West	0.94 (0.87 - 1.02)	.15

[a]Total N = 23,811 with 12,481 events

© 2022 Pichert MD et al. *JAMA Network Open.*

eTable 7. Cox Proportional Hazards Model of Patients who Received Chemotherapy and Radiation with Immunotherapy for Stage III NSCLC with Time to Immunotherapy within the PACIFIC Trial Limits and Outside the PACIFIC Trial Limits as a Covariate.

	**Hazard Ratio (95% CI)**	**P value**
Time to Immunotherapy		
≤2 weeks	Reference	
3-6 Weeks	1.40 (0.79 - 2.49)	.25
>6 Weeks	1.27 (0.72 - 2.24)	.41
Age (yrs)		
20-49	Reference	
50-64	1.17 (0.68 - 2.01)	.57
65-74	0.98 (0.55 - 1.77)	.96
≥75	1.01 (0.54 - 1.87)	.98
Sex		
Male	Reference	
Female	0.85 (0.70 - 1.04)	.12
Race		
White	Reference	
Black	0.75 (0.54 - 1.04)	.08
Asian	0.29 (0.10 - 0.79)	.02
Ethnicity		
Non-Hispanic	Reference	
Hispanic	0.78 (0.37 - 1.63)	.50
Insurance		
Private	Reference	
Medicare	1.11 (0.83 - 1.47)	.48
Medicaid	0.94 (0.65 - 1.37)	.76
Uninsured	1.17 (0.60 - 2.29)	.64
Other Government	0.75 (0.37 - 1.52)	.42
Median Income		
<$38,000	Reference	
$38,000-$47,999	0.84 (0.60 - 1.17)	.29
≥$48,000	0.71 (0.53 - 0.96)	.03
Year of Diagnosis		
2015	Reference	
2016	0.98 (0.60 - 1.57)	.92
2017	0.73 (0.50 - 1.06)	.10

eTable 7 Continued.

Charlson-Deyo Score		
0	Reference	
1	1.13 (0.90 - 1.41)	.30
2	1.49 (1.09 - 2.04)	.01
3	1.52 (1.04 - 2.22)	.03
Histology		
Adenocarcinoma	Reference	
Squamous Cell	1.51 (1.23 - 1.86)	<.001
Large Cell	2.00 (0.78 - 5.13)	.15
Other	0.73 (0.48 - 1.12)	.15
T Category		
1	Reference	
2	1.34 (0.98 - 1.83)	.07
3	1.50 (1.08 - 2.09)	.02
4	1.58 (1.12 - 2.22)	.01
N Category		
0	Reference	
1	0.88 (0.49 - 1.56)	.66
2	1.23 (0.80 - 1.91)	.34
3	1.71 (1.07 - 2.73)	.02
Facility Type		
Non-Academic	Reference	
Academic	0.79 (0.63 - 0.98)	.03
Region		
Northeast	Reference	
Midwest	0.97 (0.73 - 1.29)	.84
South	0.94 (0.71 - 1.25)	.67
West	0.87 (0.59 - 1.27)	.46

[a]Total N = 1,297 with 439 events

© 2022 Pichert MD et al. *JAMA Network Open.*

eTable 8. Previously Reported Hazard Ratios for Overall Survival Comparing Durvalumab Plus Chemoradiation to Chemoradiation Alone.

Study	Hazard Ratio	P Value
Desilets et al.[1]	0.56 (0.37 – 0.85)	.001
Sankar et al.[2]	0.57 (0.50 – 0.66)	<.001
Fukui et al.[3]	0.68 (0.45 – 0.997)	.003

References

1. Desilets A, Blanc-Durand F, Lau S, et al. Durvalumab therapy following chemoradiation compared with a historical cohort treated with chemoradiation alone in patients with stage III non-small cell lung cancer: A real-world multicentre study. *Eur J Cancer.* 2021;142:83-91.
2. Sankar K, Bryant AK, Strohbehn GW, et al. Real World Outcomes versus Clinical Trial Results of Durvalumab Maintenance in Veterans with Stage III Non-Small Cell Lung Cancer. *Cancers.* 2022;14(3).
3. Fukui T, Hosotani S, Soda I, et al. Current status and progress of concurrent chemoradiotherapy in patients with locally advanced non-small cell lung cancer prior to the approval of durvalumab. *Thorac Cancer.* 2020;11(4):1005-1014.

© 2022 Pichert MD et al. *JAMA Network Open.*

eTable 9. P-Values Before and After Benjamini-Hochberg Multiple Testing Adjustment

	Hazard Ratio	Unadjusted p-value	P-value with Benjamini-Hochberg Correction Applied
Male	0.71 (0.63-0.81)	0.0001	0.0003
White	0.75 (0.68-0.84)	0.0001	0.0003
Non-Hispanic	0.74 (0.67-0.82)	0.0001	0.0003
Medicare	0.69 (0.6-0.79)	0.0001	0.0003
Income ≥$48,000	0.68 (0.6-0.78)	0.0001	0.0003
CDS 0	0.72 (0.63-0.82)	0.0001	0.0003
Academic	0.71 (0.61-0.84)	0.0001	0.0003
Non-Academic	0.76 (0.67-0.85)	0.0001	0.0003
Age 65-74	0.68 (0.58-0.79)	0.0001	0.0003
Age ≥75 yr	0.61 (0.48-0.76)	0.0001	0.0003
Clinical N2	0.72 (0.63-0.82)	0.0001	0.0003
Stage IIIA	0.7 (0.61-0.81)	0.0001	0.0003
Non-Squamous	0.67 (0.58-0.77)	0.0001	0.0003
Clinical T3	0.69 (0.56-0.85)	0.0004	0.0011
Clinical T4	0.73 (0.61-0.87)	0.0006	0.0016
CDS 1	0.74 (0.62-0.88)	0.0007	0.0018
Northeast	0.72 (0.58-0.88)	0.0014	0.0033
West	0.62 (0.46-0.84)	0.0017	0.0038
Female	0.8 (0.69-0.92)	0.0019	0.004
Stage IIIB	0.79 (0.68-0.92)	0.002	0.004
Midwest	0.77 (0.64-0.91)	0.0025	0.0048
South	0.78 (0.67-0.92)	0.0035	0.0064
Squamous	0.82 (0.71-0.94)	0.0038	0.0066
Clinical T2	0.79 (0.66-0.94)	0.0066	0.011
Income $38,000-$47,999	0.77 (0.63-0.94)	0.01	0.016
Medicaid	0.69 (0.5-0.94)	0.0197	0.0303
Clinical T1	0.75 (0.59-0.97)	0.0252	0.0373
Clinical N3	0.8 (0.66-0.98)	0.0269	0.0384
Asian	0.3 (0.1-0.92)	0.0353	0.0487
Clinical N1	0.67 (0.44-1.01)	0.0543	0.0724
Black	0.76 (0.57-1.01)	0.0582	0.0751
Age 50-64	0.88 (0.76-1.02)	0.0943	0.1179
CDS 3	1.36 (0.94-1.97)	0.0995	0.1206
Private Insurance	0.88 (0.75-1.04)	0.1364	0.1605
Clinical N0	0.77 (0.52-1.14)	0.1875	0.2143
Income <$38,000	0.9 (0.7-1.16)	0.4074	0.4527
Age <50 yr	0.85 (0.5-1.44)	0.5414	0.5853
CDS 2	0.91 (0.67-1.24)	0.5571	0.5864
Hispanic	0.87 (0.43-1.79)	0.7099	0.7281
Uninsured	0.91 (0.45-1.82)	0.7836	0.7836
